# Spanish version of the ICIQ-Bowel questionnaire among colorectal cancer patients: construct and criterion validity: Comprehensive assessment of bowel function

**DOI:** 10.1186/s12876-023-02970-6

**Published:** 2023-10-09

**Authors:** Cinara Sacomori, Luz Alejandra Lorca, Mónica Martinez-Mardones, Marta Natalia Pizarro-Hinojosa, Gonzalo Sebastián Rebolledo-Diaz, Jessica Andrea Vivallos-González

**Affiliations:** 1 Facultad de Medicina Clínica Alemana, Universidad del Desarrollo, Avenida Plaza 680, Las Condes, Kinesiología, Santiago de, Chile; 2 Hospital del Salvador, Servicio de Medicina Física y Rehabilitación, Santiago de, Chile; 3 Hospital del Salvador, Servicio de Cirugía y Servicio de Coloproctología, Santiago de, Chile; 4 Escuela de Medicina, Universidad Finis Terrae, Santiago de, Chile

**Keywords:** Bowel function, Questionnaire, Colorectal Cancer, Anorectal function

## Abstract

**Purpose:**

Bowel complaints are very common among patients with colorectal cancer. However, the most used questionnaires for colorectal cancer survivors do not comprehensively comprise bowel symptoms. This study aimed to examine construct and criterion validity, as well as internal consistency, of the Chilean Version of the International Consultation on Incontinence Questionnaire Bowel Module (ICIQ-B) among people with colorectal cancer.

**Methods:**

Cross-sectional, validation study performed with 106 colorectal cancer patients from Hospital del Salvador, Chile. Bowel function was assessed with the ICIQ-B. Construct validity was assessed with confirmatory factor analysis and hypothesis testing. Specific items of a quality-of-life questionnaire (EORTC QLQ-CR29) were used to correlate with similar ICIQ-B items for criterion validity. For internal consistency, Cronbach’s alpha was computed.

**Results:**

For construct validity, the confirmatory factor analysis showed that the three factors model did not fit our data. Meanwhile, hypothesis testing favored the construct validity of the instrument, considering that rectal cancer patients showed worse bowel pattern (p = 0.001), bowel control (p = 0.001) and quality of life (p < 0.001) scores compared to colon cancer patients. In addition, those patients assessed before surgery also presented worse scores bowel control (p = 0.023) and quality of life (p = 0.009) compared to post-surgical patients. Regarding criterion validity, the ICIQ-B items showed a significant correlation with similar QLQ-CR29 items. The internal reliability of the instrument was good (Cronbach’s α = 0.909).

**Conclusion:**

Considering that this questionnaire appraises bowel function in more depth, it is recommended for use in clinical practice and research with colorectal cancer patients.

**Supplementary Information:**

The online version contains supplementary material available at 10.1186/s12876-023-02970-6.

## Introduction

Colorectal cancer is a persistent challenge for health professionals due to its high incidence and mortality rates worldwide. One study analyzing trends from databases of 39 countries concluded that the incidence of colorectal cancer has continued to increase in countries with medium-to-high human development indices, as well as in younger populations [1]. However, due to increasing incidence and survival rates, improvements in quality of life have become more relevant. With advanced treatment techniques, fewer patients require a long-term stoma, and bowel function is considered an important outcome for colorectal cancer patients [2].

Patients with colorectal cancer may manifest bowel symptoms in many stages of the natural evolution of the disease and with all treatments. A systematic review showed that one supportive care need of colorectal cancer patients was to get more information about the long-term self-management of symptoms and complications at home, such as bowel symptoms [3]. A common symptom of colorectal cancer is the change in bowel habits, and is considered an important factor in its diagnosis [4]. Bowel dysfunction has been exhaustively described following low anterior resection for rectal cancer repair and is known as low anterior resection syndrome (LARS). This syndrome is characterized mainly by incontinence, high stool frequency, fecal urgency, dysfunction related to fecal content elimination, problems with gas–stool discrimination, and negative impact on quality of life [5]. Bowel symptoms similar to LARS have also been reported after sigmoid resection in approximately 40% of patients [6].

Recent studies have demonstrated that colon cancer patients, after surgery, also present bowel symptoms similar to LARS [6–8]. A recent case-control study identified that serious LARS was present in 52% of rectal cancer patients; meanwhile, sigmoid cancer patients showed a prevalence of LARS symptoms similar to a non-operative control population at risk of developing colorectal cancer (around 25%) [8]. An Australian study showed that bladder and bowel symptoms were worse in colorectal cancer patients than in the population norms both pre- and postoperatively [9]. Some aggravating factors for bowel symptoms are being female, having previously had a temporary stoma [7], radiotherapy [10, 11], tumor height, and low anterior resection [11].

Continuous assessment of bowel function in this population is needed in order to prevent functional impairment and to plan rehabilitation. Many instruments have been used for such purposes. A recent study with rectal cancer survivors in a watch and wait program, i.e. treated only with chemoradiotherapy, found a discrepancy in bowel symptoms prevalence between LARS score and Wexner score [12]. According to the authors, this inconsistency shows the lack of a validated instrument to assess dysfunction following non-surgical treatment of rectal cancer [12].

The International Consultation on Incontinence Questionnaire Anal Incontinence Symptoms and Quality of Life Module (ICIQ-B) is an instrument for learning about bowel symptoms that has some advantages compared to other questionnaires for anorectal symptoms. This questionnaire includes a comprehensive assessment of bowel control, bowel pattern, and impact on quality of life. Coterill et al. (2011) reported on the psychometric properties of the original English version. They assessed a large sample of people with anal incontinence [13]. Sacomori et al. (2021) adapted the ICIQ-B to Spanish and evaluated its face validity and test and re-test reliability among Chilean colorectal cancer patients [14]. But there is still a need to test other measurement properties of the Chilean version of the ICIQ-B. Accordingly, this study aimed to examine construct and criterion validity and internal consistency of the Chilean Version of the ICIQ-B among colorectal cancer patients. We hypothesized that the Chilean version of ICIQ-B would be a robust instrument considering the psychometric proposed tests. More specifically, regarding hypothesis testing construct validity, we expected that rectal cancer patients (compared to colon), those assessed at pre-surgery (compared to post-surgery) and women (compared to men) would present worse bowel symptoms.

## Methods

This cross-sectional validation study used the terminology and reporting guidelines of COSMIN [15]. All participants gave their written informed consent, and the study was approved by the Ethical Committee of Servicio de Salud Metropolitano Oriente [approval on 28th May 2019], Santiago de Chile. The questionnaire results were collected on paper by four previously trained physical therapists that asked patients about their preference on the form of administration: self-completion or interviewed-assisted.

### Participants

This study included 106 patients diagnosed with colorectal cancer at a public hospital in Santiago, Chile: Hospital del Salvador. Patients were invited to participate when attending medical checkups either before or up to 10 years of survival after surgical cancer treatment. None refused to participate. The data collection was carried out between October 2019 and May 2022 with a delay due to the Covid-19 pandemics. The exclusion criteria were people with stoma, cognitive deficit, or illiteracy (without sufficient understanding of Spanish), people under 18 years-old, or with neurological conditions that might interfere in their bowel function.

Prior sample size calculation estimated a total sample of 105 participants, considering that Hospital del Salvador annually cares for around 180 colorectal cancer patients, and the recommendation of a reliable sample of at least 5 patients for each item of the questionnaire [16].

### Instruments

The ICIQ-B assesses comprehensively bowel and anal incontinence symptoms (including fecal and gas incontinence) and their impact on quality of life. Each item has a bowel symptom frequency scale and a visual numeric scale assessing the bothersome of the symptoms, but the last is not considered to compute scores. The instrument has 21 items separated into three domains with scores from 1 to 21 for bowel patterns (sum of 5 items), 0–28 for bowel control (sum of 7 items) and 0–26 for quality of life related to bowel symptoms (sum of 5 items). In addition, it has four unscored items related to other bowel symptoms, including the Bristol stool consistency scale. The response scale for most of the items is: never, rarely, some of the time, most of the time and always. Developed in United Kingdom, the English version of the questionnaire has proven to be robust and psychometrically solid, considering analysis of content, construct, criterion validity, internal consistency, and reliability [13, 17]. Its Spanish version has shown to be appropriate regarding face validity and test-retest reliability, available at Supplementary File 1 [14]. The full English version can be found at ICIQ website (https://iciq.net/wp-content/uploads/2019/08/Sample-ICIQ-B.pdf).

The European Organization for Research and Treatment of Cancer Quality of Life Questionnaire complement specific for colorectal cancer (EORTC QLQ CR29) was used for criterion validity through concurrent analysis of the ICIQ-B and similar items of the EORTC QLQ CR29. The EORTC QLQ- CR29 evaluates pelvic floor symptoms but places more emphasis in quality of life. Each item has a scale from 1 to 4 (1 = not at all, 2 = a little, 3 = quite a bit, 4 = very much). A scoring manual is available to compute the scores for each domain, which could vary from 0 to 100; with 100 representing better functioning or worse symptoms [18]. It addresses gastrointestinal symptoms (stool frequency, bloating, flatulence, fecal incontinence, blood or mucus in stool, dry mouth, taste), pain (abdominal pain, buttock pain, dyspareunia, sore skin), problems with micturition (urinary frequency and incontinence, dysuria), psychosocial aspects (body image, hair loss, anxiety, weight, embarrassment, sexual interest, impotence) and stoma care problems. This questionnaire was selected instead of other bowel symptoms questionnaires (Wexner or FIQL) because, similarly to the ICIQ-B, it has a focus on quality of life, and it is more representative of colorectal cancer symptoms.

Additional sociodemographic and clinical information was obtained from clinical records, including age, marital status, education level, type of cancer, previous cancer treatments, body mass index, and comorbidities (diabetes, hypertension, depression/anxiety, and respiratory and musculoskeletal problems).

### Data analysis

The data were analyzed with SPSS® version 20.0 (SPSS Inc., Chicago, Illinois, 2011). Descriptive statistics was used to characterize participants. Data normality was checked with the Kolmogorov Smirnov test. There was missing data only for characteristics of the participants, so no imputation was required.

Construct validity was firstly assessed with confirmatory factor analysis performed with AMOS® using the maximum likelihood estimation. It tested the three domains structure of ICIQ-B. The stand-alone items “Other bowel symptoms” were not considered for factor analysis [13]. We followed the proposed criteria for model fit assessment: goodness-of-fit test (p > 0.15 indicating a good fit), Steiger’s root mean square error of approximation (RMSEA, p < 0.05 indicating a good fit and an upper value of 0.08 representing a reasonable fit), comparative fit index (CFI, expected to be > 0.90 to indicate a good fit) and the non-normed fit index (NNFI, expected to be > 0.90 to indicate a good fit) [19]. Secondly, an, hypothesis testing was performed comparing bowel symptoms between groups (Mann Whitney test): type of cancer (colon or rectal), stage of treatment (pre- or post-surgery), and gender. We hypothesized that rectal cancer patients (compared to colon), those assessed at pre-surgery (compared to post-surgery) and women (compared to men) would present worse bowel symptoms.

Spearman correlations were computed between the scores of each domain of the ICIQ-B and the EORTC QLQ-CR29 score to estimate criterion validity. The concordance was studied between similar items (i.e., that assessed the same symptom) of the ICIQ-B and the EORTC QLQ-CR29, respectively: items 3a and 4a with the Stool Frequency scale, item 7a with the Sore Skin scale, items 9a and 10a with the Fecal Incontinence scale, item 11a with the Flatulence scale, item 19a with the Embarrassment scale, and item 18a with the Sexual Interest scale. Correlation coefficients of < 0.49 were defined as poor, 0.50 ≤ rho ≤ 0.74 as fair, and rho > 0.75 as a strong relationship.

For internal consistency, the Cronbach’s alpha test was used, and values higher than 0.70 were considered to show good internal consistency [16]. A p < 0.05 was set for all tests.

## Results

### Participants characteristics

The mean age of the 106 participants was 67.6 (SD = 12.4) years and the mean body mass index was 25.45 (SD = 4.4). Most were female (61.3%) and retired (44%) (Table 1). Regarding their clinical characteristics, most had colon cancer (71.7%), but only 42 participants (40%) were assessed post-surgery (Mean = 32.3 months, SD = 32.5). Body weight excess was present in 49.4% and hypertension in 51.4% of the participants.

The prevalence of liquid stool incontinence was 43.4% (n = 46), solid stool incontinence was 32.1% (n = 34), and gas incontinence was 60.4% (n = 64). Urgency to defecate was present in 52.8% (n = 56) of the patients. Most of them reported defecating between one and three times a day (68.9%, n = 73), while 19.8% (n = 21) had to defecate three to ten times a day, and 2.8% (n = 3) reported ten times or more a day. During sleeping hours only 24.5% (n = 26) of the patients reported having to defecate.

**Table 1:** Sociodemographic and clinical characteristics of participants (n = 106)

Variable	n (%)
Sex	
Female Male	65 (61.3) 41 (38.7)
Work status*	
Stable contract Independent Unemployed Retired	23 (23.0) 17 (17.0) 16 (16.0) 44 (44.0)
Schooling level*	
Elementary (basic) education Secondary Technical University	15 (14.9) 44 (43.6) 20 (19.8) 22 (21.8)
Marital status*	
Married Stable union Divorced Single	45 (44.1) 1 (1) 12 (11.7) 23 (22.5)
Type of cancer	
Colon Rectum	76 (71.7) 30 (28.3)
Received cancer treatments	
Chemotherapy Radiotherapy Surgery	42 (39.6) 26 (24.5) 42 (40.0)
Body weight classification*	
Underweight Healthy weight range Overweight Obesity	0 (0) 48 (50.5) 33 (34.7) 14 (14.7)
Comorbidities	
Respiratory problems Depression/anxiety Musculoskeletal problems Diabetes Mellitus 2 Arterial hypertension	16 (15.1) 39 (36.8) 37 (34.9) 16 (15.2) 54 (51.4)
Regular use of laxative	12 (11.3)
Regular use of antidiarrheal drugs	5 (4.7)

*Valid percent was used as there were missing cases.

### Construct validity

**Fig. 1:**
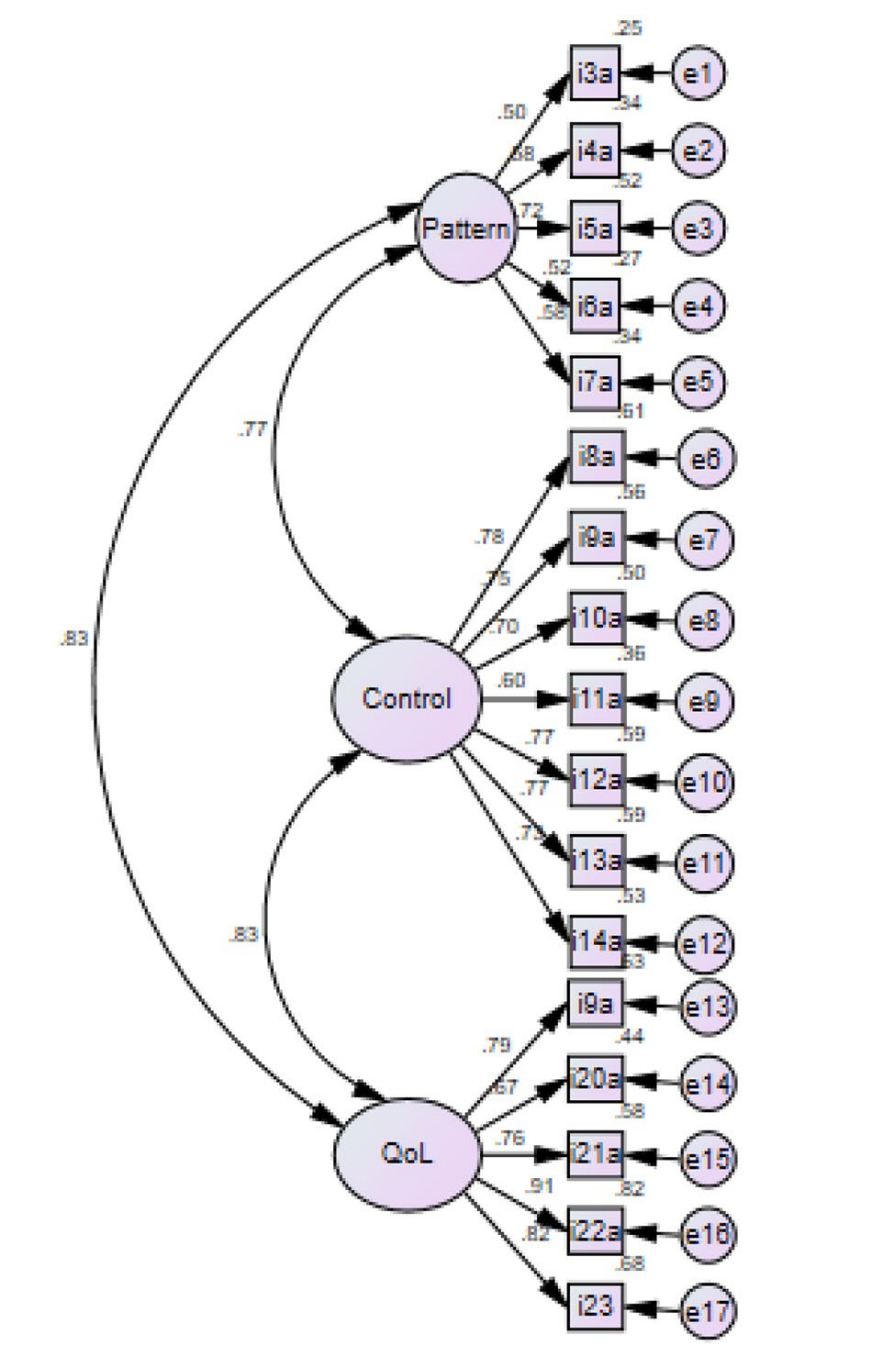
Confirmatory factor analysis of the three-factor model: bowel pattern, bowel control and quality of life (QoL)

Results of the confirmatory factor analysis are presented in Fig. 1. Factor loadings, representing the relationship among the items and domains, were mostly high (≥ 0.7); except for the items 3a (evacuation frequency), 4a (evacuation frequency at night), 6a (taking antidiarrheal drugs), 7a (pain/soreness around anus), 11a (gas incontinence) and their respective factors, which had factor loadings ≤ 0.6. However, the model did not show adequate fit for our data [*X*^2^ goodness-of-fit test (df = 162) = 327.78, p < 0.001; RMSEA = 0.09; NNFI = 0.805 and CFI = 0.898).

Hypothesis tests were performed comparing ICIQ-B scores considering type of cancer, gender, and surgical treatment. We identified that rectal cancer patients showed significantly higher scores in all ICIQ-B domains compared to colon cancer patients (Table 2). In addition, patients assessed prior to cancer surgery had significantly higher scores for the ICIQ-B domains bowel control (p = 0.023) and quality of life (p = 0.009) when compared to those assessed post-surgery. Meanwhile, bowel pattern scores were similar both before and after surgery (p = 0.232). There was no significant difference between men and women regarding bowel pattern (p = 0.849), bowel control (p = 0.995) or quality of life (p = 0.433).

**Table 2:** Bowel symptoms scores, overall and according to cancer type (n = 106)

Domain	All (n = 106)	Colon (n = 76)	Rectal (n = 30)	p*
	Md (IR)	Md (IR)	Md (IR)	
Bowel pattern	4 (4.3)	3.5 (4)	6 (6.3)	0.001
Bowel control	3 (9)	2 (6)	7.5 (13.5)	0.001
Quality of life related to bowel function	4 (11)	2 (8)	11.5 (12.3)	< 0.001

*Mann Whitney test. Md = median. IR = interquartile range

We identified a floor effect for most of the variables (except question 3a related to the defecation frequency).

### Criterion validity

The QLQ stool frequency scale was significantly correlated to ICIQ-B items 3a (rho = 0.540, p < 0.001) and 4a (rho = 0.600, p < 0.001). Sore skin scale correlated with item 7a (rho = 0.523, p < 0.001). Fecal incontinence scale correlated with items 9a (rho = 0.521, p < 0.001) and 10a (rho = 0.352, p < 0.001). Flatulence scale correlated with item 11a (rho = 0.591, p < 0.001). Embarrassment scale correlated with item 19a (rho = 0.498, p < 0.001). Finally, Sexual Interest scale correlated with item 18a (rho=-0.402, p < 0.001).

### Internal consistency

The internal reliability of the instrument for the total scale, excluding Bristol scale for stool consistency as recommended [13], was good (Cronbach’s α = 0.909). The internal consistency for domains bowel pattern (α = 0.710), bowel control (α = 0.879), and quality of life (α = 0.817) was good.

## Discussion

This study demonstrated that the Spanish version of the ICIQ-B is appropriate for clinical use among colorectal cancer patients as our results suggested adequate values compared to a criterion and good internal consistency. Regarding its construct validity, the confirmatory factor analysis did not support the fit of the three-factor model to our data. These results might be explained by the characteristics of our sample which was not composed of only incontinent individuals but of people before and after receiving aggressive cancer treatments. Patients before and after colorectal cancer treatment may present a variety of bowel symptoms, from constipation to diarrhea and incontinence [2–4]. All these symptoms can be assessed individually with ICIQ-B items. Possibly, a more homogenous sample, selecting only rectal or colon cancer patients after cancer treatment would be more appropriate to test the three domains structure of the instrument.

Another indicator for construct validity used in this study, the hypothesis testing, was mostly fulfilled. We confirmed two of the three previous hypotheses: (1) rectal cancer patients showed worse bowel function compared to colon cancer patients, and (2) bowel function prior to surgery was worse compared to post-surgery. However, our third hypothesis was not confirmed as there was no difference between men and women regarding bowel domains.

As expected, rectal cancer patients had worse bowel function compared to colon cancer patients. Rectal cancer and its treatments affect anorectal function more than colon cancer. After colon cancer surgeries, diarrhea is common and more liquid stools increase the risk of incontinence [2]. After sphincter preserving surgeries for rectal cancer, bowel disfunctions are very common presenting with incontinence, urgency and increased frequency of opening the bowels [5, 20]. Preoperative chemoradiotherapy also increases the risk of showing bowel dysfunctions after rectal cancer surgery [10].

Before cancer surgery, our participants showed worse bowel control and quality of life compared to those participants assessed post treatments. Similarly, another study with 30 colorectal cancer patients from Australia, using the ICIQ-B, showed that the percentage of participants with bowel symptoms decreased from 60% preoperatively to 44% 6 months after surgery [21]. The previous study also highlights that at six months after surgery, patients had significantly less abdominal pain but more fecal incontinence compared to their preoperative status [21]. The difference regarding fecal incontinence comparing to our study might be attributed to our assessment after surgery that was not standardized and was quite longer (Mean = 32.3 months after cancer surgery) compared to the previous study.

Bowel symptoms were present both before and after treatments, the most prevalent were liquid stool incontinence (43.4%), gas incontinence (60.4%), and urgency to defecate (52.8%). Sometimes colorectal cancer survivors are not aware of these bowel habit changes. A qualitative study demonstrated that patients would like to have received information, previous to their colorectal cancer treatments, regarding possible changes in bowel habit and impact on diet [22]. After treatments, colorectal cancer survivors experience survivorship as an individual, life-changing process, with uncertainties and need to deal with bowel dysfunction and ostomy [23].

Contrary to what was expected, men and women from this study had similar scores for bowel function and quality of life. At least one year after colorectal cancer surgery, being female is a risk factor for presenting worse bowel dysfunction [7]. The incidence of major LARS was bigger among women (18%) compared to men (9%) [24]. Differently to the previously mentioned studies, ours also included people assessed before cancer treatments, which could justify why there was no gender differences. It is well-known that non-treated colorectal cancer manifests with a variety of bowel symptoms for both men and women [4].

In the criterion validity analysis, we found a fair correlation between items of the ICIQ-B and the EORTC QLQ CR29, which is acceptable. However, a study with rectal cancer patients after sphincter preserving surgery found that neither the EORTC C-30 nor the CR-38 are sensitive instruments in delineating differences in bowel function [25]. It is very common to use quality-of-life questionnaires to assess bowel function but using more specific questionnaires such as the ICIQ-B is probably more appropriate. Future studies should compare the ICIQ-B with other scores for bowel symptoms such as Wexner, Fecal Incontinence Quality of Life Scale (FIQL), and LARS score.

Internal consistency was good for the total set of question items (α = 0.909) and for the domains of bowel pattern (α = 0.710), bowel control (α = 0.879), and quality of life (α = 0.817). These values were similar to the English original version [13]. Likewise, the identified tendency to a floor effect might be explained as this questionnaire was originally developed to assess the bowel function among anal incontinent individuals.

The LARS terminology is broadly used and, sometimes, studies also use this terminology and the LARS score indiscriminately for other types of surgeries, such as those for colon cancer [6]. We suggest that the better terminology to use is bowel dysfunctions, anorectal symptoms, or anorectal dysfunctions. For such purposes, the ICIQ-B is a comprehensive instrument that considers the complete framework of bowel function that might be useful for diagnosing dysfunctions, as well as planning and confirming the effectiveness of treatments.

The limitations of this study were mainly related to not including only incontinent patients or those post cancer treatment, which was justified by the need to measure bowel function as a broader concept both before and after colorectal cancer treatments. Another limitation was not adding another measure like ICIQ-B questionnaire for criterion validity. Data collection took much longer than planned due to the Covid-19 pandemics. As a strength, this is one of the first studies to report data on bowel function of Latin-American colorectal cancer patients. We suggest that upcoming studies aiming to test ICIQ-B factorial structure include higher samples sizes and a more diversified sample, i.e., people with fecal incontinence symptoms not necessarily related to colorectal cancer.

In conclusion, although we did not confirm the three domains structure of the ICIQ-B Chilean version with colorectal cancer patients, the questionnaire presented robust psychometric properties regarding internal consistency, construct validity throughout hypothesis testing and criterion validity. Considering that ICIQ-B appraises bowel function in more depth, it could be a valuable instrument for many clinicians that assist colorectal cancer patients. The results of this study confirm that ICIQ-B is a questionnaire suitable for use among people with colorectal cancer as it exhaustively represents the variety of bowel symptoms they face along the continuum of care. Accordingly, we strongly recommend the use of ICIQ-B questionnaire for clinical practice and research because it is one of the most comprehensive instruments to deeply understand bowel symptoms. One of its advantages is that it includes the assessment of fecal urgency and frequency of evacuations, which are relevant for people living with and beyond colorectal cancer. The limitation of ICIQ-B is that it is quite longer than traditionally used questionnaires (Wexner, Vaisey, LARS score). Future studies might aim to provide a short version of this questionnaire.

### Electronic supplementary material

Below is the link to the electronic supplementary material.

Supplementary Material 1

Supplementary Material 2

## Data Availability

Data will be available upon request to corresponding author e-mail.
