# Supplementary material for: Spanish version of the ICIQ-Bowel questionnaire among colorectal cancer patients: construct and criterion validity: Comprehensive assessment of bowel function
Source: BMC Gastroenterol. 2023 Oct 9;23:352. doi: 10.1186/s12876-023-02970-6 (PMC10563276; doi:10.1186/s12876-023-02970-6)
Supplement: Supplementary file 1 — Supplementary Material 1 [file 12876_2023_2970_MOESM1_ESM.pdf]

ICIQ-B (04/08)

Numero Inicial

CONFIDENCIAL

DIA MES AÑO

Día de hoy

Muchas personas experimentan accidentes o escapes intestinales. Estamos intentando descubrir cuántas personas experimentan estos síntomas y cuánto las molesta. Agradeceríamos si pudiera responder las siguientes preguntas, en relación a cómo ha estado usted en LOS ÚLTIMOS TRES MESES.

1 Por favor, escriba su fecha de nacimiento:

DIA MES AÑO

2 ¿Cuál es su sexo?: Femenino ☐ Masculino ☐

B Patrón Intestinal

3 En promedio, ¿cuántas veces va usted al baño a defecar dentro de 24 horas?
(Marque una alternativa para "usualmente" y otra para "cuando está peor")

	**(a) Usualmente**	(b) **Cuando está peor**
menos de una vez	☐ 1	☐ 1
una a tres veces	☐ 2	☐ 2
tres a diez veces	☐ 3	☐ 3
diez veces o más	☐ 4	☐ 4

(c) **¿Cuánto le molesta esto?**
Por favor, elija un número de 0 (nada) a 10 (muchísimo)

0 1 2 3 4 5 6 7 8 9 **10**
nada muchísimo

4 ¿Con qué frecuencia va a defecar durante la noche? desde que se acuesta hasta que se levanta en la mañana. *(Elija una alternativa)* (a)

nunca	☐ 0
una vez	☐ 1
dos veces	☐ 2
tres veces	☐ 3
Cuatro o más veces	☐ 4

(b) **¿Cuánto le molesta esto?**
Por favor, elija un número de 0 (nada) a 10 (muchísimo)

0 1 2 3 4 5 6 7 8 9 **10**
nada muchísimo

SAMPLE

Copyright © "ICIQ group"

5 **¿Tiene usted que acudir al baño corriendo con apuro, cuando tiene ganas de defecar?** *(Elija una alternativa)*

(a)

nunca	☐	0
rara vez	☐	1
algunas veces	☐	2
la mayoría de las veces	☐	3
siempre	☐	4

¿Cuánto le molesta esto?
Por favor, elija un número de 0 (nada) a 10 (muchísimo)

0 1 2 3 4 5 6 7 8 9 **10**
nada muchísimo

6 **¿Usa algún medicamento (pastilla o líquido) para detener la defecación?** *(Elija una alternativa)*

(a)

nunca	☐	0
menos de una vez al mes	☐	1
menos de una vez a la semana	☐	2
menos de una vez al día	☐	3
como una vez al día	☐	4
varias veces al día	☐	5

(b) **¿Cuánto le molesta esto?**
Por favor, elija un número de 0 (nada) a 10 (muchísimo)

0 1 2 3 4 5 6 7 8 9 **10**
nada muchísimo

7 **¿Ha sentido dolor/sensibilidad alrededor del ano?** *(Elija una alternativa)*

(a)

nunca	☐	0
rara vez	☐	1
algunas veces	☐	2
la mayoría de las veces	☐	3
siempre	☐	4

(b) **¿Cuánto le molesta esto?**
Por favor, elija un número de 0 (nada) a 10 (muchísimo)

0 1 2 3 4 5 6 7 8 9 **10**
nada muchísimo

Puntaje de patrón intestinal: suma de puntajes 3a - 7a ☐ ☐

Copyright © “ICIQ group”

Control Intestinal

8 **¿Mancha la ropa interior o ha necesitado usar compresas protectoras debido a su defecación?** *(Elija una alternativa)*

(a)

nunca	☐	0
menos de una vez al mes	☐	1
menos de una vez a la semana	☐	2
Menos de una vez al día	☐	3
todos los días	☐	4

(b) **¿Cuánto le molesta esto?**
Por favor, elija un número de 0 (nada) a 10 (muchísimo)

0 1 2 3 4 5 6 7 8 9 **10**
nada muchísimo

9 **¿Es capaz de controlar la pérdida de deposiciones líquidas o semilíquidas a través del ano?** *(Elija una alternativa)* (a)

siempre	☐	0
mayoría de las veces	☐	1
algunas veces	☐	2
rara vez	☐	3
nunca	☐	4

(b) **¿Cuánto le molesta esto?**
Por favor, elija un número de 0 (nada) a 10 (muchísimo)

0 1 2 3 4 5 6 7 8 9 **10**
nada muchísimo

10 **¿Es capaz de controlar pérdidas accidentales de deposiciones sólidas o formadas a través del ano?** *(Elija una alternativa)*

(a)

siempre	☐	0
mayoría de las veces	☐	1
algunas veces	☐	2
rara vez	☐	3
nunca	☐	4

(b) **¿Cuánto le molesta esto?**
Por favor, elija un número de 0 (nada) a 10 (muchísimo)

0 1 2 3 4 5 6 7 8 9 **10**
nada muchísimo

SAMPLE

Copyright © "ICIQ group"

11 **¿Es capaz de controlar el escape de gases a través del ano?**
(Elija una alternativa)

(a)

siempre	☐	0
mayoría de las veces	☐	1
algunas veces	☐	2
rara vez	☐	3
nunca	☐	4

(b) **¿Cuánto le molesta esto?**
Por favor, elija un número de 0 (nada) a 10 (muchísimo)

0 1 2 3 4 5 6 7 8 9 **10**
nada muchísimo

12 **¿Es capaz de controlar el escape de mucosidad (secreción) a través del ano?** *(Elija una alternativa)*

(a)

siempre	☐	0
mayoría de las veces	☐	1
algunas veces	☐	2
rara vez	☐	3
____ nunca	☐	4

(b) **¿Cuánto le molesta esto?**
Por favor, elija un número de 0 (nada) a 10 (muchísimo)

0 1 2 3 4 5 6 7 8 9 **10**
nada muchísimo

13 **¿Ha tenido pérdidas o escape accidental de deposiciones sin tener ganas de ir al baño?** *(Elija una alternativa)*

(a)

nunca	☐	0
rara vez	☐	1
algunas veces	☐	2
mayoría de las veces	☐	3
____ siempre	☐	4

(b) **¿Cuánto le molesta esto?**
Por favor, elija un número de 0 (nada) a 10 (muchísimo)

0 1 2 3 4 5 6 7 8 9 **10**
nada muchísimo

SAMPLE

Copyright © "ICIQ group"

14 ¿Son los accidentes o escapes de deposiciones impredecibles?
(Elija una alternativa)

(a)

nunca	☐	0
rara vez	☐	1
algunas veces	☐	2
mayoría de las veces	☐	3
siempre	☐	4

(b) **¿Cuánto le molesta esto?**
Por favor, elija un número de 0 (nada) a 10 (muchísimo)

0 1 2 3 4 5 6 7 8 9 **10**
nada muchísimo

Puntaje de control intestinal: suma de los puntajes 8a – 14a ☐ ☐

Otros síntomas intestinales

15 Usando las figuras, por favor indique ¿Cómo son sus heces la mayor parte del tiempo? *(Elija todas las alternativas que aplican)*

(a)

trozos duros separados, como nueces (difícil de pasar) 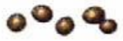 ☐ 1

con forma de salchicha, pero grumosa 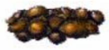 ☐ 2

con forma de salchicha, pero con grietas 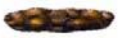 ☐ 3

1 con forma de salchicha, como serpiente lisa y suave 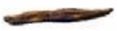 ☐ 4

bolas suaves con bordes desiguales (fácil de pasar) 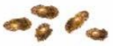 ☐ 5

trozos suaves con los bordes desiguales, heces como pulpa 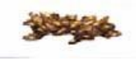 ☐ 6

acuosa, sin trozos sólidos (totalmente líquida) 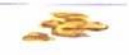 ☐ 7

(b) **¿Cuánto le molesta esto?**
Por favor, elija un número de 0 (nada) a 10 (muchísimo)

0 1 2 3 4 5 6 7 8 9 **10**
nada muchísimo

SAMPLE

Copyright © "ICIQ group"

Bristol Stool Form Scale O'Donnell, L.J.D. et al (1990)

16 ¿Necesita hacer fuerza (pujar) para eliminar las deposiciones?
(Elija una alternativa)

(a)

nunca ☐ 0
rara vez ☐ 1
algunas veces ☐ 2
mayoría de las veces ☐ 3
siempre ☐ 4

(b) **¿Cuánto le molesta esto?**
Por favor, elija un número de 0 (nada) a 10 (muchísimo)

0 1 2 3 4 5 6 7 8 9 **10**
nada muchísimo

17 ¿Piensas en la posibilidad de tener algún escape accidental de deposiciones? *(Elija una alternativa)*

(a)

nunca ☐ 0
rara vez ☐ 1
algunas veces ☐ 2
mayoría de las veces ☐ 3
siempre ☐ 4

(b) **¿Cuánto le molesta esto?**
Por favor, elija un número de 0 (nada) a 10 (muchísimo)

0 1 2 3 4 5 6 7 8 9 **10**
nada muchísimo

Impacto sexual

18 ¿Restringe usted su actividad sexual debido a sus deposiciones?
(Elija una alternativa)

(a)

nunca ☐ 0
rara vez ☐ 1
algunas veces ☐ 2
mayoría de las veces ☐ 3
siempre ☐ 4
no aplica ☐ 5

(b) **¿Cuánto le molesta esto?**
Por favor, elija un número de 0 (nada) a 10 (muchísimo)

0 1 2 3 4 5 6 7 8 9 **10**
nada muchísimo

SAMPLE

Copyright © “ICIQ group”

Calidad de Vida

19 ¿Se siente avergonzado debido a sus deposiciones?
(Elija una alternativa)

(a)

nunca ☐ 0
rara vez ☐ 1
algunas veces ☐ 2
mayoría de las veces ☐ 3
siempre ☐ 4

(b) **¿Cuánto le molesta esto?**
Por favor, elija un número de 0 (nada) a 10 (muchísimo)

0 1 2 3 4 5 6 7 8 9 **10**
nada muchísimo

20 ¿Está pendiente de dónde está el baño debido a sus deposiciones?
(Elija una alternativa)

(a)

nunca ☐ 0
rara vez ☐ 1
algunas veces ☐ 2
mayoría de las veces ☐ 3
____ siempre ☐ 4

(b) **¿Cuánto le molesta esto?**
Por favor, elija un número de 0 (nada) a 10 (muchísimo)

0 1 2 3 4 5 6 7 8 9 **10**
nada muchísimo

21 ¿Sus deposiciones causan que usted haga planes de acuerdo a estas? *(Elija una alternativa)*

(a)

nunca ☐ 0
rara vez ☐ 1
algunas veces ☐ 2
mayoría de las veces ☐ 3
siempre ☐ 4

(b) **¿Cuánto le molesta esto?**
Por favor, elija un número de 0 (nada) a 10 (muchísimo)

0 1 2 3 4 5 6 7 8 9 **10**
nada muchísimo

0 1 2 3 4 5 6 7 8 9 **10**
nada muchísimo

SAMPLE

Copyright © "ICIQ group"

22 **¿Sus deposiciones lo obliga a quedarse en la casa más seguido de lo que a usted le gustaría?** *(Elija una alternativa)*

(a)

nunca	☐	0
rara vez	☐	1
algunas veces	☐	2
mayoría de las veces	☐	3
siempre	☐	4

(b) **¿Cuánto le molesta esto?**
Por favor, elija un número de 0 (nada) a 10 (muchísimo)

0 1 2 3 4 5 6 7 8 9 **10**
nada muchísimo

23. En general, ¿cuánto interfieren sus deposiciones con su vida diaria?

Por favor elija un número de 0 (nada) a 10 (muchísimo)

0 1 2 3 4 5 6 7 8 9 **10**
nada muchísimo

Puntaje de calidad de vida: suma de los puntajes 19a -23 ☐ ☐

24 **Por favor, en el espacio abajo describa cualquier otra preocupación sobre tener escapes o accidentes de sus deposiciones, qué piensa podría haber causado su pérdida de deposiciones, o algo más que deberíamos saber.**

Muchas gracias por contestar estas preguntas.

SAMPLE

Copyright © "ICIQ group"
