# Supplementary material for: Spanish version of the ICIQ-Bowel questionnaire among colorectal cancer patients: construct and criterion validity: Comprehensive assessment of bowel function
Source: BMC Gastroenterol. 2023 Oct 9;23:352. doi: 10.1186/s12876-023-02970-6 (PMC10563276; doi:10.1186/s12876-023-02970-6)
Supplement: Supplementary file 2 — Supplementary Material 2 [file 12876_2023_2970_MOESM2_ESM.docx]

Link to access the full English version of ICIQ-B

<https://iciq.net/wp-content/uploads/2019/08/Sample-ICIQ-B.pdf>
